# Supplementary material for: Dietary diversity, nutritional status and associated factors among lactating mothers visiting government health facilities at Dessie town, Amhara region, Ethiopia
Source: PLoS One. 2022 Feb 17;17(2):e0263957. doi: 10.1371/journal.pone.0263957 (PMC8853554; doi:10.1371/journal.pone.0263957)
Supplement: S1 Data — (DOC) [file pone.0263957.s001.doc]

**Table 1: Distribution for DDS**

| **Descriptive** | | | | |
| --- | --- | --- | --- | --- |
|  | | | Statistic | Std. Error |
| BMI | Mean | | 22.5437 | .17205 |
|  | 95% Confidence Interval for Mean | Lower Bound | 22.2055 |  |
|  |  | Upper Bound | 22.8819 |  |
|  | 5% Trimmed Mean | | 22.3705 |  |
|  | Median | | 22.5850 |  |
|  | Variance | | 12.078 |  |
|  | Std. Deviation | | 3.47533 |  |
|  | Minimum | | 16.00 |  |
|  | Maximum | | 35.38 |  |
|  | Range | | 19.38 |  |
|  | Interquartile Range | | 4.44 |  |
|  | Skewness | | .629 | .121 |
|  | Kurtosis | | .778 | .241 |

|  | **Tests of Normality** | | | | | | | | | | | | | | | |  |
| --- | --- | --- | --- | --- | --- | --- | --- | --- | --- | --- | --- | --- | --- | --- | --- | --- | --- |
|  |  | Kolmogorov-Smirnov^a^ | | | | | | | Shapiro-Wilk | | | | | | | |  |
|  |  | Statistic | | | df | | Sig. | | Statistic | | df | | | Sig. | | |  |
|  | BMI | .125 | | | 408 | | .000 | | .956 | | 408 | | | .000 | | |  |
|  | a. Lilliefors Significance Correction | | | | | | | | | | | | | | | |  |
| **Table 2. Skewness and Kurtosis result for the outcome variable, DDS**  **Descriptive Statistics** | | | | | | | | | | | | | | | | | |
|  | | | N | Minimum | | Maximum | | Mean | | Std. Deviation | | Skewness | | | Kurtosis | | |
|  |  |  | Statistic | Statistic | | Statistic | | Statistic | | Statistic | | Statistic | Std. Error | | Statistic | Std. Error | |
| BMI | | | 408 | 16.00 | | 35.38 | | 22.5437 | | 3.47533 | | .629 | .121 | | .778 | .241 | |
| Valid N (listwise) | | | 408 |  | |  | |  | |  | |  |  | |  |  | |

|  | | |
| --- | --- | --- |
| **Statistics**  BMI | | |
| N | Valid | 408 |
|  | Missing | 0 |
| Mean | | 22.5437 |
| Skewness | | .629 |
| Std. Error of Skewness | | .121 |
| Kurtosis | | .778 |
| Std. Error of Kurtosis | | .241 |


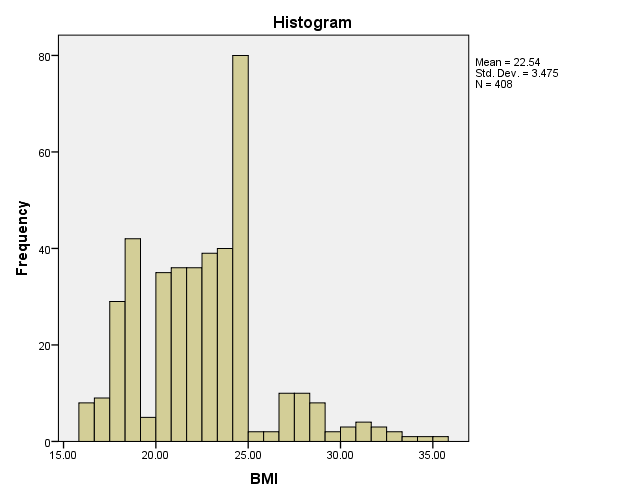


**Figure 1: Distribution Graph for Maternal Body Mass Index (BMI)**

**Table 3: Distribution for DDS**

| **Descriptive**   |  |  | | | | | | | | | | | | --- | --- | --- | --- | --- | --- | --- | --- | --- | --- | --- | --- | |  |  | | | | | | | | | | | | StatisticStd. Error | **DDS** | | | | | | | **Mean** | | 5.3235 | | | .08632 |  | | 95% Confidence Interval for Mean | | | | | Lower Bound | | 5.1538 | | |  |  | | | Upper Bound | | 5.4932 | |  | | |  | 5.3121 | |  | |  | | |  | Median | | | | | 5.0000 | |  | | |  | Variance | | | | | 3.040 | |  | | |  | Std. Deviation | | | | | 1.74357 | |  | | |  | Minimum | | | | | 1.00 | |  | | |  | Maximum | | | | | 9.00 | |  | | |  | Range | | | | | 8.00 | |  | | |  | Interquartile Range | | | | | 3.00 | |  | | |  | Skewness | | | | | .119 | | .121 | | |  | Kurtosis | | | | | -.558 | | .241 | | |  |  | | | | |  | |  | | |  | | | | | | | | | | | | | dfSig.Kolmogorov-Smirnov^a^Shapiro-Wilk | | Statistic | | | | | df | | | | | | .130 | | 408 | .000 | | .963 | | 408 | | .000 | | a. Lilliefors Significance Correction | |  | |  |  | |  | |  | |  | |  | | | | | | | | | | | |   **Table 4. Skewness and Kurtosis result for the outcome variable (DDS)** | | | | | | | | | | | | |
| --- | --- | --- | --- | --- | --- | --- | --- | --- | --- | --- | --- | --- |
|  | N | Minimum | | | Maximum | | Mean | Std. Deviation | Skewness | | Kurtosis | |
|  | Statistic | Statistic | | | Statistic | | Statistic | Statistic | Statistic | Std. Error | Statistic | Std. Error |
| DDS | 408 | 1.00 | | | 9.00 | | 5.3235 | 1.74357 | .119 | .121 | -.558 | .241 |
| Valid N (listwise) | 408 |  | | |  | |  |  |  |  |  |  |
|  |  | | | | |  | | | | | | |
|  | **Statistics**  **DDS** | | | | |  | | | | | | |
|  | N | | Valid | 408 | |  | | | | | | |
|  |  |  | Missing | 0 | |  | | | | | | |
|  | Mean | | | 5.3235 | |  | | | | | | |
|  | Median | | | 5.0000 | |  | | | | | | |
|  | Mode | | | 5.00 | |  | | | | | | |
|  | Std. Deviation | | | 1.74357 | |  | | | | | | |
|  | Skewness | | | .119 | |  | | | | | | |
|  | Std. Error of Skewness | | | .121 | |  | | | | | | |
|  | Kurtosis | | | -.558 | |  | | | | | | |
|  | Std. Error of Kurtosis | | | .241 | |  | | | | | | |


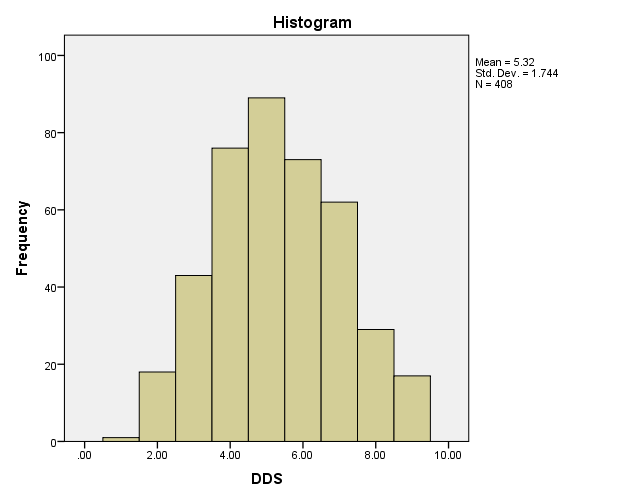


**Figure 2. Distribution Graph for Maternal Dietary Diversity Scores (DDS)**

**Table 5.Correlation of BMI with other variables**

**Decision for assessing if a test is significant; If *p*≤.05: significant and if *p*>.05** not significant

| **Correlations** | | | |
| --- | --- | --- | --- |
|  | | BMI | Age |
| BMI | Pearson Correlation | 1 | .327^**^ |
|  | Sig. (2-tailed) |  | .000 |
|  | N | 408 | 408 |
| Age | Pearson Correlation | .327^**^ | 1 |
|  | Sig. (2-tailed) | .000 |  |
|  | N | 408 | 408 |
|  |  | BMI | Maternal Educational status |
| BMI | Pearson Correlation  Sig. (2-tailed)  N | 1  408 | .116^*^  .019  408 |
| Maternal Educational status | Pearson Correlation  Sig. (2-tailed)  N | .116^*^  .019  408 | 1  408 |
|  |  | BMI | Paternal Education |
| BMI | Pearson Correlation  Sig. (2-tailed)  N | 1  408 | .163^**^  .001  408 |
| Paternal Education | Pearson Correlation  Sig. (2-tailed)  N | .163^**^  .001  408 | 1  408 |
|  |  | BMI | Household monthly income |
| BMI | Pearson Correlation  Sig. (2-tailed)  N | 1  408 | .165^**^  .001  408 |
| Household monthly income | Pearson Correlation  Sig. (2-tailed)  N | .165^**^  .001  408 | 1  408 |
|  |  | BMI | Head of household |
| BMI | Pearson Correlation  Sig. (2-tailed)  N | 1  408 | .175^**^  .000  408 |
| Head of household | Pearson Correlation  Sig. (2-tailed)  N | .175^**^  .000  408 | 1  408 |
|  |  | BMI | Family size |
| BMI | Pearson Correlation  Sig. (2-tailed)  N | 1  408 | .218^**^  .000  408 |
| Family size | Pearson Correlation  Sig. (2-tailed)  N | .218^**^  .000  408 | 1  408 |
|  |  | BMI | Food security |
| BMI | Pearson Correlation  Sig. (2-tailed)  N | 1  408 | -.281^**^  .000  408 |
| Food security | Pearson Correlation  Sig. (2-tailed)  N | -.281^**^  .000  408 | 1  408 |
|  |  | BMI | Women DDS |
| BMI | Pearson Correlation  Sig. (2-tailed)  N | 1  408 | -.239^**^  .000  408 |
| Women DDS | Pearson Correlation  Sig. (2-tailed)  N | -.239^**^  .000  408 | 1  408 |
|  |  | BMI | Type of House |
| BMI | Pearson Correlation  Sig. (2-tailed)  N | 1  408 | .142^**^  .004  408 |
| Type of House | Pearson Correlation  Sig. (2-tailed)  N | .142^**^  .004  408 | 1  408 |
|  |  | BMI | Marital status |
| BMI | Pearson Correlation  Sig. (2-tailed)  N | 1  408 | -.060  .225  408 |
| Marital status | Pearson Correlation  Sig. (2-tailed)  N | -.060  .225  408 | 1  408 |
| *. Correlation is significant at the 0.05 level (2-tailed).  **. Correlation is significant at the 0.01 level (2-tailed). | | | |

**Table 6: Correlation of DDS with independent variables**

**Decision for assessing if a test is significant; If *p*≤.05: significant and if *p*>.05 not significant**

| **Correlations** | | | |
| --- | --- | --- | --- |
|  | | Women DDS | Maternal_Education |
| Women DDS | Pearson Correlation | 1 | -.148^**^ |
|  | Sig. (2-tailed) |  | .003 |
|  | N | 408 | 408 |
| Maternal Education | Pearson Correlation | -.148^**^ | 1 |
|  | Sig. (2-tailed) | .003 |  |
|  | N | 408 | 408 |
|  |  | Women DDS | Paternal Education |
| Women DDS | Pearson Correlation  Sig. (2-tailed)  N | 1  408 | -.131^**^  .008  408 |
| Paternal Education | Pearson Correlation  Sig. (2-tailed)  N | -.131^**^  .008  408 | 1  408 |
|  |  | Women DDS | HH monthly income |
| Women DDS | Pearson Correlation  Sig. (2-tailed)  N | 1  408 | -.244^**^  .000  408 |
| HH monthly income | Pearson Correlation  Sig. (2-tailed)  N | -.244^**^  .000  408 | 1  408 |
|  |  | Women DDS | Nutrition information |
| Women DDS | Pearson Correlation  Sig. (2-tailed)  N | 1  408 | .142^**^  .004  408 |
| Nutrition information | Pearson Correlation  Sig. (2-tailed)  N | .142^**^  .004  408 | 1  408 |
|  |  | Women DDS | Avoidance of food during lactation |
| Women DDS | Pearson Correlation  Sig. (2-tailed)  N | 1  408 | .005  .926  408 |
| Avoidance of food during lactation | Pearson Correlation  Sig. (2-tailed)  N | .005  .926  408 | 1  408 |
|  |  | Women DDS | Changes in food intake |
| Women DDS | Pearson Correlation  Sig. (2-tailed)  N | 1  408 | .113^*^  .022  408 |
| Changes in food intake | Pearson Correlation  Sig. (2-tailed)  N | .113^*^  .022  408 | 1  408 |
|  |  | Women DDS | BMI |
| Women DDS | Pearson Correlation  Sig. (2-tailed)  N | 1  408 | -.219^**^  .000  408 |
| BMI | Pearson Correlation  Sig. (2-tailed)  N | -.219^**^  .000  408 | 1  408 |
|  |  | Women DDS | Maternal Age_ |
| Women DDS | Pearson Correlation  Sig. (2-tailed)  N | 1  408 | .078  .117  408 |
| Maternal Age | Pearson Correlation  Sig. (2-tailed)  N | 078  .117  408 | 1  408 |
|  |  | Women DDS | Food security status |
| Women DDS | Pearson Correlation  Sig. (2-tailed)  N | 1  408 | .204^**^  .000  408 |
| Food security status | Pearson Correlation  Sig. (2-tailed)  N | .204^**^  .000  408 | 1  408 |
|  |  | Women DDS | Type of House |
| Women DDS | Pearson Correlation  Sig. (2-tailed)  N | 1  408 | -.187^**^  .000  408 |
| Type of House | Pearson Correlation  Sig. (2-tailed)  N | -.187^**^  .000  408 | 1  408 |
| **. Correlation is significant at the 0.01 level (2-tailed).  *. Correlation is significant at the 0.05 level (2-tailed). | | | |
