# Supplementary material for: Dietary diversity, nutritional status and associated factors among lactating mothers visiting government health facilities at Dessie town, Amhara region, Ethiopia
Source: PLoS One. 2022 Feb 17;17(2):e0263957. doi: 10.1371/journal.pone.0263957 (PMC8853554; doi:10.1371/journal.pone.0263957)
Supplement: S1 Questionnaire — (DOC) [file pone.0263957.s003.doc]

# ANNEX I: INFORMATION AND CONSENT SHEET

**Bahir Dar Institute of Technology, Bahir Dar University**

Faculty of Chemical and Food Engineering

|  |
| --- |
| **Information and Consent sheet**  Hello, my name is ___________________ I am working as a data collector with Awel Seid, who is completing his master’s Degree in Applied Human Nutrition, at Bahir Dar University,Bahir Dar Institute of Technology. He is conducting a study on “Dietary Diversity, Nutritional Status and Associated Factors among Lactating Women Visiting Governmental Health Facilities of Dessie Town”. This study is, therefore, part of the requirements for the fulfillment of the MSc programme he is enrolled in.  We will ask you questions about your health and household.Your participation in this study is voluntary and you may stop the interview at any time when you got bored. The interview will take just 30 minutes. In additionwe will also measure your weight and height. The data we collect from you will be entirely confidentialand your answers will not be given to anyone rather they will be used only for purpose of this research. Your name will not alsobe identified in the report. We would like to have complete data but you do not have to answer questions you do not want to. However, I hope that you will participate in this study since your views are important.  Finally, I would like to assure that there is no any risk that would be happens by involving in the study. For detail information you can contact the investigator through cell phone: 0961499705 and e-mail awelseid03@gmail.com.   - If you have any questions about the survey I am ready to respond. Do you have any questions? - Please let me know if anything I have stated is not clear and I will be happy to explain it further to ensure you understand.   I have been informed about the purpose and use of this particular research. After all these I understood and:  1. I agree to participate in this research voluntarily  2. I didn’t agree to participate in this research  Interviewer name __________________________ Signature_________Date ____________  ***Thank you in advance for your cooperation to the study!*** |

# ANNEX II: ENGLISH VERSION QUESTIONNAIRE

Name of the interviewer __________________________ Date of interview _____________

**Date of visit** [_____|_____| ______| **CODE** _____________

DD |MM |YYYY

| **Part I፡ Background &Socio-demographic characteristics information about the lactating woman** | | | | | | | | | | |
| --- | --- | --- | --- | --- | --- | --- | --- | --- | --- | --- |
| N^O^ | | **Questions** | | **Alternatives/Answers** | | | | | | **Skip Guide** |
| 101 | | How old are you? | | Age in complete years ___________ | | | | | |  |
| 102 | | What is your religion? | | Muslim (1)  Orthodox (2)  Protestant (3)  Others (specify)___________ (4) | | | | | |  |
| 103 | | What is your educational status? | | No formal Education (1)  Primary Education (1-8) (2)  Secondary Education (9-12) (3)  College Diploma and above (4) | | | | | |  |
| 104 | | What is your husband’s educational status? | | No formal Education (1)  Primary Education (1-8) (2)  Secondary Education (9-12) (3)  College Diploma and above (4) | | | | | |  |
| 105 | | What is your occupation? | | House wife (1)  Daily laborer (2)  Merchant (3)  Private Business (4)  Government employee (5)  Other (specify) ____________ (6) | | | | | |  |
| 106 | | What is your husband’sOccupation? | | No work (1)  Daily laborer (2)  Merchant (3)  Private Business (4)  Government employee (5)  Other (specify) ____________ (6) | | | | | |  |
| 107 | | How much is your family monthly income? | | ______________ ETB. | | | | | |  |
| 108 | | What type of house do you live in? | | Grass roof/hut (1)  Corrugated iron roof with walls made of soil (2)  Corrugated iron roof with walls made of cement (3) | | | | | |  |
| 109 | | Who is the head of your household? | | Husband (1)  Wife (2)  Both husband and wife (3) | | | | | |  |
| 110 | | What is your current marital status? | | Married/Living together (1)  Single (never married) (2)  Divorced/ Separated (3)  Widowed (4) | | | | | |  |
| **Part II. Questions on obstetric history and health service utilization of lactating women** | | | | | | | | | | |
| 201 | | Have you face any illness in the previous two weeks? | | | Yes (1)  No (0) | | | | |  |
| 202 | | Have you got any type of nutrition education? | | | Yes (1)  No (0) | | | | | If the answer is no skip to 301 |
| 203 | | If the answer to Q-209is yes, from where did you get it? | | | From health workers (1)  From mass media (3)  Others (specify) __________ (4) | | | | |  |
| **Part III: Questions related to dietary diversity of lactating women**  Please describe the foods (meals and snacks) that you ate yesterday during the day and night, whether at home or outside the home. Start with the first food eaten in the morning.  Enumerator: Please probe carefully about the food items that the mother ate yesterday during the day and night, whether at home or outside the home. | | | | | | | | | | |
|  | | **Food group consumed in previous 24 hour** | **Description/examples** | | | | | | **Response Yes (1) No (0)** | |
| 301 | | Starchy staples, roots and tuber crops | Breads, porridges of maize, sorghum, millet or cassava, rice, pasta/noodles potatoes, white-fleshed sweet potatoes | | | | | |  | |
| 302 | | Dark green leafy vegetables | Any medium-to-dark green leafy vegetables, including, Broccoli, Chili greens, cabbage, Kale, Lettuce, Spinach | | | | | |  | |
| 303 | | Other vitamin A rich fruits and vegetables | Ripe mango, ripe papaya, orange fleshed sweet potato, carrot, pumpkin, pepper. | | | | | |  | |
| 304 | | Other fruits and vegetables | Apple, Avocado, Banana, Grapes, Guava, Lemon, Mandarin, Strawberry, Lettuce, Cucumbers, Onion, Peas(when eaten as fresh pod), Eggplant, Corn, Green pepper, Tomato, Zucchini. | | | | | |  | |
| 305 | | Fats and oils | Any fat and oils | | | | | |  | |
| 306 | | Meat and fish | All meats, organ meats (liver, kidney, heart), Beef, lamb, goat poultry,fresh and dried fish | | | | | |  | |
| 307 | | Eggs | Any egg and foods with eggs | | | | | |  | |
| 308 | | Legumes, nuts and seeds | Bean, kidney bean, chickpea, pigeon pea, lentil and soybean/soybean products or other legume products. Seeds: sunflower seed, Pumpkin seed, groundnut/peanut, or peanut butter, Flaxseed seed etc. | | | | | |  | |
| 309 | | Milk and milk products | Milk, cheese, yoghurt or other milk products rather than butter | | | | | |  | |
| **Part IV፡ Questions related to eating habits of lactating women during lactation** | | | | | | | | | | |
| 401 | | How many times did you eat dailyin the previous 7 days? | | | | Two times (1)  Three times (2)  Four times and more (3) | |  | | |
| 402 | | Did you avoid eating any food during lactation? | | | | Yes (1)  No (2) | |  | | |
| 403 | | Did you change food intake during lactation? | | | | Yes (1)  No (2) | | If the answer is no skip to 405 | | |
| 404 | | If the answer is yes to question 404, what were your food intake changes? | | | | Frequency of meal (1)  Amount of meal (2)  Both frequency & amount of meal (3) | |  | | |
| **Part V: Questions to assess household food security condition (HFIAS9 items)** | | | | | | | | | | |
|  | **Questions** | | | | | | | **Response Yes (1)**  **No (0)** | | |
| 501 | In the past four weeks, did you worry that your household would not have enough food? | | | | | | |  | | |
| 502 | In the past four weeks, were you or any household member not able to eat the kinds of foods you preferred because of a lack of resources? | | | | | | |  | | |
| 503 | In the past four weeks, did you or any household member have to eat a limited variety of foods due to a lack of resources? | | | | | | |  | | |
| 504 | In the past four weeks, did you or any household member forced to eat un preferred foods because of a lack of resources to obtain other types of food? | | | | | | |  | | |
| 505 | In the past four weeks, did you or any household member have to eat a smaller portion of meals than you felt you needed because there was not enough food? | | | | | | |  | | |
| 506 | In the past four weeks, did you or any household member skip meals due to lack of food? | | | | | | |  | | |
| 507 | In the past four weeks, was there ever no food to eat of any kind in your household because of lack of resources to get food? | | | | | | |  | | |
| 508 | In the past four weeks, did you or any household member go to sleep at night hungry because there was not enough food? | | | | | | |  | | |
| 509 | In the past four weeks, did you or any household member go a whole day and night without eating anything because there was not enough food? | | | | | | |  | | |
| **Part VI: Anthropometric measurement of lactating women** | | | | | | | | | | |
| 601 | | Weight in Kg | | | | | **_______ Kg** | | | |
| 602 | | Height in Cm | | | | | **______ Cm** | | | |
|  | |  | | | | |  | | | |

***THANK YOU FOR YOUR GENUINE E RESPONSE TO MY QUESTIONS!***
