# Supplementary material for: Dietary diversity, nutritional status and associated factors among lactating mothers visiting government health facilities at Dessie town, Amhara region, Ethiopia
Source: PLoS One. 2022 Feb 17;17(2):e0263957. doi: 10.1371/journal.pone.0263957 (PMC8853554; doi:10.1371/journal.pone.0263957)
Supplement: Attachment — Submitted filename: Comments.docx [file pone.0263957.s004.docx]

**Comments:**

1. It is important that this study highlights the important issues faced by lactating mothers, especially in resource poor context. The manuscript will therefore be of interest to those who are working in the related fields, and I hope can be published in the PLOS ONE after a revision.
2. Background: The authors have attempted to highlight the importance of food security and dietary diversity. However, I could not find a clear rationale why the authors wanted to focus on both the outcomes at the same time in this manuscript. In addition, it would be helpful if the authors state the scientific gaps and how this study is going to fill the gaps. Why do the authors think that this study is beneficial for researchers and the public in other regions within and outside the country? Further, the authors have mentioned “lactating mothers” often, but did not explain what they mean by “lactating mothers” in this study.
3. Line 56-57- reference is missing?
4. Why author mentioned about health service system in line 60-63? How this statements link to the study objectives?
5. Line 67-69, when these study were conducted?
6. Provide theoretical basis for selecting independent variables in the study? What theories underpin your study?
7. The HFIAS tools was validated in Tanzania and Iran, how would you justify the use of this tool in Ethiopia?
8. What are the possible bias in the study and how it was attempted to minimize it?
9. Why study participants were categorized only into two levels of food-security 123 status (food-secured and food-insecure)? Why not the cumulative HFIAS score was categorized into four levels of household food insecurity: food secured, and mild, moderate, and severe food insecurity, following HFIAS guideline.
10. What does nutrition information refers to?
11. What happen after pretesting of tools?
12. Line 136-38, what is your reference to take this cut of value for low and high DDS.
13. “For mothers with age below 18years, BMI for age was calculated” what reference you used for this measurement?
14. Why all independent variables were fitted in to a multivariate logistic regression model to identify factors associated with dependent variables? Why not only significant variables?
15. In table 1, need to mention what does” P” “a” “b” stands for , and where does it come from?
16. Also, the p-value 0.00 need to be presented in standard form for writing p-value.
17. P-value in all tables need to be presented in standard format.
18. Digit after decimal need to be uniform though out the manuscript.
19. Discussion need to focus on major findings of the study and also please revise this section thoroughly and provide sufficient discussion of relevant studies.
20. Please also check this study: “Food insecurity and dietary diversity among lactating mothers in the urban municipality in the mountains of Nepal”. <https://journals.plos.org/plosone/article/authors?id=10.1371/journal.pone.0227873>
21. Language correction is required. I suggest author to have proof read of the manuscript from native English language speaker.
22. Could you please also provide your data set for review purpose?
