# Supplementary material for: Dietary diversity, nutritional status and associated factors among lactating mothers visiting government health facilities at Dessie town, Amhara region, Ethiopia
Source: PLoS One. 2022 Feb 17;17(2):e0263957. doi: 10.1371/journal.pone.0263957 (PMC8853554; doi:10.1371/journal.pone.0263957)
Supplement: Attachment — Submitted filename: Response to reviewers.doc [file pone.0263957.s005.doc]

**Authors’ response to reviewers’ comments**

Manuscript title: Dietary diversity, nutritional status and associated factors among lactating mothers visiting government health facilities at Dessie town, Amhara region, Ethiopia

| **Reviewer** | **Reviewer’s comments** | **Authors’ response** |
| --- | --- | --- |
| **Reviewer-1** | 1. Background: The authors have attempted to highlight the importance of food security and dietary diversity. However, I could not find a clear rationale why the authors wanted to focus on both the outcomes at the same time in this manuscript.  In addition, it would be helpful if the authors state the scientific gaps and how this study is going to fill the gaps.    Why do the authors think that this study is beneficial for researchers and the public in other regions within and outside the country?  Further, the authors have mentioned “lactating mothers” often, but did not explain what they mean by “lactating mothers” in this study. | Thank you for this comment. The main focus of this study is to determine the dietary diversity and nutritional status of lactating women. In other words, the outcome variables are dietary diversity and nutritional status. Here, food security is considered as an independent variable.  Thank you so much for this valuable comment. Factors associated with dietary diversity and nutritional status may vary from setting to setting. Besides information about dietary diversity and nutritional status of lactating mothers in Ethiopia is scant. This study was thus designed to fill these scientific gaps. This is now included in the manuscript.  Thank you for this comment. The result will be important for other regions in the country or other developing countries which have similar settings with our study area/population. This is now included in the manuscript text as well.  Thank you for the comment. Lactating mothers are those mothers who had a breastfeeding child and were nursing their breast milk for their children during the study period. This is now included in the manuscript text. |
|  | 2. Line 56-57- reference is missing? | Thank you so much for the comment. The paragraph is a bit modified now to make it more related to our topic as per the request of the second reviewer. |
|  | 4. Line 67-69, when these study were conducted? | Thank you for the comment. The studies were conducted from 2011-2016. This is now included in the manuscript text. |
|  | 5.Provide theoretical basis for selecting independent variables in the study? What theories underpin your study? | Thank you for this comment. We have read several literatures (on factors/ independent variables associated with dietary diversity and nutritional status of lactating mothers) and developed a conceptual/ theoretical framework based on these literatures. |
|  | 6.The HFIAS tools was validated in Tanzania and Iran, how would you justify the use of this tool in Ethiopia? | Thank you so much for this critical comment. This tool was also validated in Ethiopia in 2015 with a recommendation of modifying some wordings and adaptations in using the tool. We have now included this reference in the manuscript text. |
|  | 7.What are the possible bias in the study and how it was attempted to minimize it? | We used 24 hour dietary recall to determine the dietary diversity of lactating women and this might incur recall bias. We tried to minimize this bias by repeated probing. |
|  | 8.Why study participants were categorized only into two levels of food-security status (food-secured and food-insecure)? Why not the cumulative HFIAS score was categorized into four levels of household food insecurity: food secured, and mild, moderate, and severe food insecurity, following HFIAS guideline. | Thank you so much for this comment. We determined the food security status as food secure and insecure since food security was an independent variable in our study and we assumed this information is enough to tell whether there is an association with our outcome variables or not. Had food security been our outcome variable, we would have determined the food security status into four levels. |
|  | 9. What does nutrition information refers to? | Thank you so much for this comment. Nutrition information refers to having and or obtaining any information related to nutrition (including feeding during pregnancy and lactation, consumption of diversified food items, inclusion of fruit and vegetables in the diet, micronutrient supplementation etc.) This is now explained in the manuscript text. |
|  | 10. What happen after pretesting of tools? | Thank you for this comment. The result of the pretest indicated that the tool had no major problems and the tool was used as it is for the actual data collection. |
|  | 11. Line 136-38, what is your reference to take this cut of value for low and high DDS. | Thank you for this comment. While working our study, there were no established cut-off points in terms of the number of food groups to indicate adequate or inadequate dietary diversity for individual DDS. FAO (2013) suggests taking mean dietary diversity scores as cut off point and in this case those who score below the mean are considered as having inadequate dietary diversity and those who score above the mean as having adequate dietary diversity. |
|  | 12. “For mothers with age below 18years, BMI for age was calculated” what reference you used for this measurement? | Thank you for the comment. We used WHO 2007 Reference for the measurement.  (<https://cdn.who.int/media/docs/default-source/child-growth/growth-reference-5-19-years/bmi-for-age-(5-19-years)/cht-bmifa-girls-z-5-19years.pdf?sfvrsn=c708a56b_4)> |
|  | 13. Why all independent variables were fitted in to a multivariate logistic regression model to identify factors associated with dependent variables? Why not only significant variables? | Thank you for this comment. We have now included variables which showed association in the bivariate analysis. Please see the multivariate tables now. |
|  | 14. In table 1, need to mention what does” P” “a” “b” stands for , and where does it come from? | Thank you so much for this comment. They were missed in the footnote. However, based on the second reviewer’s comments, we have now modified the tables and the need for presenting the similarity or difference between means (which “a” and “b” were intended to show) is now removed. |
|  | 15. Also, the p-value 0.00 need to be presented in standard form for writing p-value. | Thank you so much for this comment. This is now addressed with changing the way we presented our data based on the other reviewer’s comments. |
|  | 16. P-value in all tables need to be presented in standard format. Digit after decimal need to be uniform though out the manuscript. | Thank you for this comment. We have now made all the decimals uniform. |
|  | 17. Discussion need to focus on major findings of the study and also please revise this section thoroughly and provide sufficient discussion of relevant studies. | Thank you for this comment. We have now improved the discussion by emphasizing on our major findings. |
|  | 18. Please also check this study: “Food insecurity and dietary diversity among lactating mothers in the urban municipality in the mountains of Nepal”. <https://journals.plos.org/plosone/article/authors?id=10.1371/journal.pone.0227873> | Thank you so much for this suggestion. We found this study very useful while revising our manuscript. We also cited it to discuss one of our results. |
|  | 19. Language correction is required. I suggest author to have proof read of the manuscript from native English language speaker. | Thank you for this comment. We have now revised the language as much as possible. |
|  | 20. Could you please also provide your data set for review purpose? | Thanks for asking this. The data set is now submitted as a supplementary file together with the manuscript. |
| **Reviewer -2** | 1.The author can explain the activities carried out by lactating women when visiting health facilities, whether the health facility provides education and counseling services during breastfeeding (so we can assume participants are healthy people) or treated for an illness. However, if the participant is sick, the conclusions in the text need to be explained more specifically that the importance of this paper is (for example) to improve the quality of services and education in health facilities, not to the public. | Thank you for this comment. Lactating women in our study refers those women who were visiting the health facilities to get vaccination services for their children and to get family planning services. All lactating women interviewed in our study were apparently healthy. This is now explained in the text. |
|  | **2. Research purposes:**  In line 77-79: “However, these factors may not be consistent in all settings and thus call for the need for context-specific information to design and implement appropriate nutrition interventions”, this statement looks inconsistent and hard to follow. The author can explain the urgency of this paper when compared with other existing data. If it is said that the factors related to dietary diversity and nutritional status are not consistent across all settings, the authors are expected to explain why this study was carried out in a more specific context, not in general. | Thank you for this valuable comment.  The statement ‘However, these factors may not be consistent in all settings and thus call for the need for context specific information to design and implement appropriate nutrition interventions’ is now removed and we replaced this statement with another one. |
|  | **3. Dietary diversity:**  Authors need to review how to interpret DDS. For example, is DDS data normally distributed or is it necessary to use distribution data. | The data is normally distributed and we can use mean score to interpret the dietary diversity score of lactating mothers. Please see the supplementary file to see the distribution of our DDS data. |
|  | • Further insights for analyzing DDS can be found in the FAO Guideline (on REF #24, page 26-27). There are no established cut-off points in terms of the number of food groups to indicate adequate or inadequate (or low/high in this text) dietary diversity for the DDS. The author can analyze using the score data from each participant to see the correlation with other variables.  If the mean DDS used as the cut-off, this would result in a low/high proportion of around 50 per cent. However, the authors need to reconsider the results and discussions regarding the prevalence of low DDS since it cannot be compared with other populations (in line 268-273). • In line 260-266 Discussion, the authors compare DDS in studies with different maximum DDS values. Please compare something equivalent.  In line 278-282: oil and fat consumption were high (98%), it mentions due to adding a small amount of oil/fat in meal preparation. The author needs to explain whether there is a restriction of food quantities to at least 15 grams to include the food group in daily consumption. For women aged 15-49 years, dietary diversity scores were more strongly correlated with micronutrient adequacy of the diet when food quantities of approximately one tablespoon or less (<15g) were not included in the score (Arimond et al., 2010). | Thank you for this comment. Correlation between DDS with each independent variable is now provided as supplementary file.  Thank you so much for this invaluable comment. We have now modified this section and compared only the means rather than proportions of populations with adequate and inadequate dietary diversity.  Thank you so much for this critical comment. The assumption is that, if the participants add oils/fats at least three times daily in their meals, this amount will add up to 15grams or more and can be considered as a food group which can contribute to their dietary diversity and in turn micronutrient adequacy. |
|  | **4. Nutritional status:** • The author needs to review whether the BMI data is normally distributed to be presented as a mean.    • On line 193-194, it says there are 21% underweight and 12% overweight. This needs to be clarified because, in Table 2 and Table 4, all participants are categorized as underweight and normal.  The author can also mention whether there are participants who fall into the obese category.  In addition, similar to DDS, the authors need to consider analyzing the correlation using the continuous variable (BMI itself) compared to the analysis after being categorized | Thank you for this comment. We have checked whether the BMI data is normally distributed or not. A histogram showing the normal distribution of the BMI data is now included as a supplementary data for further information.  Thank you for this comment. In order to apply 2-category outcome logistic model, the overweight/obese category was combined with normal BMI category. We have now clarified this in the table by putting both normal and overweight/obese (as Normal/Overweight/Obese) in the multivariate table.  Thank you for this comment. We have now included women who are in the obese category as well.  Thank you for this comment. Correlation between BMI with each independent variable is now provided as supplementary data. |
|  | **5. Dietary assessment method:** • Based on the level of the objective of dietary assessment, the authors need to explain whether the data were collected by single or replicated in non-consecutive days.  • In addition, it is necessary to clarify the method mentioned (24h dietary recall) to record all food consumed by the mother for 24 hours or recall the specific consumption of 9 food groups. | The data were collected once in a normal day which means a day without any special occasion.  Mothers were asked to recall freely what they consumed the previous day, inside and outside their home. We then categorized the foods they consumed into the nine food groups. This is now included in the manuscript text. |
|  | **6. Data analysis:** • It is recommended that the authors present the results of the correlation analysis (r and p values) for each of the tested independent variables, as stated in lines 213-215. (This might be attached in as supplementary table).  • The variables included in the regression analysis also need to be discussed regarding aspects of biological plausibility. For example, if specific variables are tested (such as maternal religion, family size, and head of household), these variables need to be discussed in the introduction/discussion. | Thank you for this comment. We have now included the correlation analysis for both DDS and BMI as supplementary data/tables.  Thank you so much for this comment. We were able to find literature support with regard to relationship between family size and nutritional status as mentioned in our introduction section. However, we couldn’t find relationship of the other variables (maternal religion and head of household) with our dependent variables. We then decided to leave them out and reanalyzed the regression again. |
|  | **6. Writing suggestions** • In the second paragraph of the Background, the author can select only information related to the topics discussed in this paper. The author needs to reconsider the relationship between urbanization, primary-secondary-tertiary level of health care with the topic.  • Authors can use more recent DHS data (is there a 2016 edition?) to describe nutritional problems in the study area.    • Paternal or parental education?    • Tables 1 & 2, contents and headings in tables are inconsistent. The author needs to review whether the DD & nutritional status data in table 1 is needed. The same data has shown in tables 3 & 4 • Authors need to add information to the superscript “a” and “b” in data tables 1 and 2.  • Eating habits: this data appears in the result, but there is no explanation regarding the meaning of habits.   - Dietary diversity categories: low/high or adequate/inadequate?   • Discussion: In the first paragraph, the author can explain the most interesting findings or the ones that answer the main problem in the research | Thank you for this comment. This paragraph is now modified. We have tried to include information which is related to the topic only.  Thank you for this comment. It is right that the data we used is old data. However, the 2016 EDHS survey didn’t assess women’s nutritional status and thus we were obliged to use the 2011 DHS data.  Thank you so much for this comment. It is to mean educational status of the husband and it is paternal. We have now replaced it by saying husband education.  Thank you so much for this comment. We have now modified the headings and the way the tables are presented. We removed the DD and Nutritional status data from table 1 as well.  Thank you for this comment. This is now addressed by explaining the meaning of eating habits in the result section and the data regarding eating habits is also used to explain some points (nutritional status of mothers) in the discussion part of the manuscript.  Thank you for this comment. Dietary diversity is now presented as adequate and inadequate rather than low or high.  Thank you for this comment. We have now modified the discussion and started by discussing the dietary diversity of lactating women followed by their nutritional status. |
