# Supplementary material for: Dietary diversity, nutritional status and associated factors among lactating mothers visiting government health facilities at Dessie town, Amhara region, Ethiopia
Source: PLoS One. 2022 Feb 17;17(2):e0263957. doi: 10.1371/journal.pone.0263957 (PMC8853554; doi:10.1371/journal.pone.0263957)
Supplement: Attachment — Submitted filename: Authors response.doc [file pone.0263957.s006.doc]

**Authors’ response to reviewer’s comments**

|  | **Reviewer’s Comments** | **Authors’ response** |
| --- | --- | --- |
| Reviewer #4 | 1. Based on table 1, one person was single/never married! How was she lactating then? | Thank you for the comment.  In some cases, it is possible for a woman to become pregnant; have a child then become lactating without being married or without having a husband formally. The case may happen if she had an informal relationship with someone (without having a formal marriage) and then she became pregnant and lactating. In our culture, a relationship is not considered as a legal/ formal unless it is committed based on our traditional practices/ beliefs/ customs. That was the reason for being considered as single but still lactating. |
|  | 2. In table 2, please put SD for mean of dietary diversity score. | Thank you for the comment. We have now included the standard deviation of the mean dietary diversity score of our participants. |
|  | 3. For tables 3 and 4, please spell out AOR and COR at foot of the tables. | Thank you for the comment. We have now included the full terms for the Abbreviations AOR and COR. |
